# First psychotic episode as first manifestation of lyme disease: Case report

**DOI:** 10.1192/j.eurpsy.2021.2148

**Published:** 2021-08-13

**Authors:** R. Almeida Leite, M. Almeida, A. Costa, J. Alcafache, A. Mesquita

**Affiliations:** Psychiatry And Mental Health, Baixo Vouga Hospital Centre, Aveiro, Portugal

**Keywords:** lyme disease, borrelia, neuroborreliosis, psychosis

## Abstract

**Introduction:**

Lyme disease (LD) is caused by the spirochete Borrelia burgdorferi (Bb) and has been reported to be associated with various psychiatric presentations.

**Objectives:**

To report a case with LD and to highlight the importance of differential diagnosis in a first psychotic episode.

**Methods:**

Case report and non-systematic review of the literature.

**Results:**

A woman aged 31 was admitted to the psychiatric department, after a car accident with a mortal victim, due to a first psychotic episode with visual hallucinations, disorientation in time and space, persecutory and grandiosity delusions. She had a personal psychiatric history of obsessive-compulsive disorder and no previous admission to an inpatient Unit. On psychotropic drugs the condition failed to improve, and subsequently neurological symptoms developed. EEG abnormalities prompted a lumbar puncture. In the CSF a strong plasma cell reaction with atypical cells was observed. The enzyme immunoassay for Borrelia burgdorferi was positive and after treatment with penicillin the psychiatric and neurological signs and symptoms remitted. Screening assessment followed by a thorough history, comprehensive psychiatric clinical exam, review of systems, mental status exam, neurological exam and physical exam relevant to the patient’s complaints and findings with clinical judgment, pattern recognition and knowledgeable interpretation of laboratory findings facilitates diagnosis. Psychotropics and antibiotics may help improve functioning and prevent further disease progression.

**Conclusions:**

LD is relatively rare, but awareness of the association between LD and neuropsychiatric presentations can improve understanding of the causes of mental illness and result in more effective prevention, diagnosis and treatment.

**Disclosure:**

No significant relationships.

